# Cannabidiol Promotes Neuronal Differentiation Using Akt and Erk Pathways Triggered by Cb1 Signaling

**DOI:** 10.3390/molecules27175644

**Published:** 2022-09-01

**Authors:** Santino Blando, Ivana Raffaele, Luigi Chiricosta, Andrea Valeri, Agnese Gugliandolo, Serena Silvestro, Federica Pollastro, Emanuela Mazzon

**Affiliations:** 1 IRCCS Centro Neurolesi “Bonino-Pulejo”, Via Provinciale Palermo, Contrada Casazza, 98124 Messina, Italy, ,; 2 Department of Pharmaceutical Sciences, University of Eastern Piedmont, Largo Donegani 2, 28100 Novara, Italy

**Keywords:** cannabidiol, neurodifferentiation, NSC-34, retinoic acid receptor-related orphan receptors, G protein-coupled receptors

## Abstract

Recently, the scientific community has started to focus on the neurogenic potential of cannabinoids. The phytocompound cannabidiol (CBD) shows different mechanism of signaling on cannabinoid receptor 1 (CB1), depending on its concentration. In this study, we investigated if CBD may induce in vitro neuronal differentiation after treatment at 5 µM and 10 µM. For this purpose, we decided to use the spinal cord × neuroblastoma hybrid cell line (NSC-34) because of its proliferative and undifferentiated state. The messenger RNAs (mRNAs) expression profiles were tested using high-throughput sequencing technology and Western blot assay was used to determine the number of main proteins in different pathways. Interestingly, the treatment shows different genes associated with neurodifferentiation statistically significant, such as *Rbfox3,* *Tubb3*, *Pax6* and *Eno2*. The CB1 signaling pathway is responsible for neuronal differentiation at 10 µM, as suggested by the presence of p-ERK and p-AKT, but not at 5 µM. A new correlation between CBD, neurodifferentiation and retinoic acid receptor-related orphan receptors (RORs) has been observed.

## 1. Introduction

*Cannabis sativa* is a plant known since ancient times for its beneficial properties [1]. The natural products that derive from *C. sativa* consist of a group of different terpenophenolic compounds called cannabinoids. Cannabinoids represent a potential therapeutic drug by triggering the endocannabinoid system (ES) and the cannabinoid receptors signaling, which are important for the maintenance of homeostasis in the body [2]. The main receptors of the ES are cannabinoid receptors 1 (CB1) and cannabinoid receptors 2 (CB2), which belong to the family of G-protein coupled receptors (GPCRs) and play a fundamental role in many cellular processes, including neuronal survival and differentiation [3]. In particular, CB1 receptors are the most expressed GPCRs in the central nervous system, predominantly on neurons and glial cells of the brain [4].

**Figure 1:**
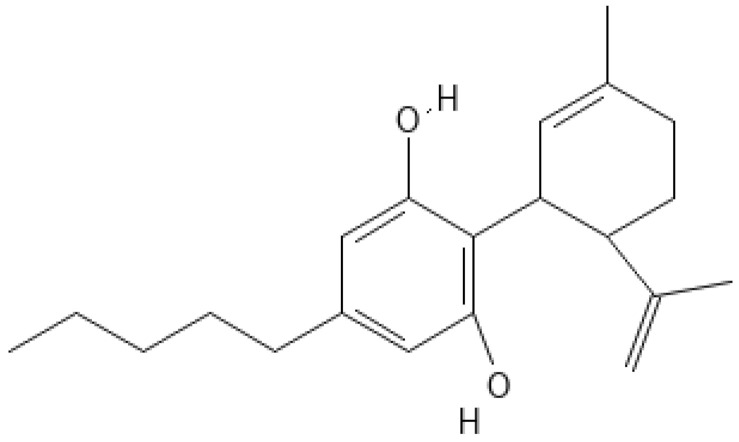
Chemical structure representation of CBD. Image obtained by Pubchem (https://pubchem.ncbi.nlm.nih.gov/compound/644019, accessed on 23 July 2022).

Previous studies have shown that the activation of CB1 regulates the neurite outgrowth followed by formation of the neurons. During this complex process, CB1 receptors are able to transduce signals to modulate gene expression and the cytoskeletal components within the cells, leading to many electrophysiological, morphological and transcriptional changes [5]. Instead, CB2 receptors are mainly expressed in immune cells and less expressed in the brain [6], but they show a similar effect to CB1 on transduction [7]. According to published data, CB1 promotes different downstream effectors using G-protein or non-G-protein partners. This occurs primarily via Gi/o and βγ subunits, affecting mostly the mitogen-activated protein kinases (MAPKs) and the phosphatidylinositide 3-kinase/protein kinase B (PI3K/AKT) pathways. However, this receptor can also act through G-protein-independent mediators, such as β-arrestins or G protein-coupled receptor kinases (GRKs) [8]. It has been shown that different agonists of CB1 stimulate varied responses in vivo; indeed, the selectivity of ligand affects the activation of distinct signaling pathways. The mechanism through which CB1 causes a switch between all the signals is poorly understood, due to the biased signaling [8,9]. Among all cannabinoids, Δ9-tetrahydrocannabinol (Δ9 -THC) and cannabidiol (CBD) are the most represented. While Δ9-THC is best known for the psychotropic effect associated with *Cannabis* consumption, CBD is non-psychoactive phytocannabinoid that shows interesting biological properties, including neuroprotective, anti-inflammatory, anti-oxidant, and anti-apoptotic effects [10,11,12]. The molecule CBD (C_21_H_30_O_2_) is a cyclohexene with a methyl group in position 1, a 2,6-dihydroxy-4-pentylphenyl group at position 3 and a prop-1-en-2-yl group at position 4 (Figure 1). According to various preclinical studies, the lack of psychotropics effects makes CBD a promising phytocompound for pharmacotherapy, due to its improved safety profile. In fact, it was demonstrated that CBD showed fewer side effects than Δ9-THC, it was not mutagenic and had low toxicity [10].

CBD has a poor affinity for CB1 and CB2 receptors. It is a negative allosteric modulator or a weak agonist of CB1, depending on its concentration [3], while it exerts an inverse agonist action on CB2. CBD exerts the action of CB1 negative allosteric modulator (NAM) at concentrations below 10 µM, while at 10 µM, it displays minimal agonist activity with the increased inhibition of Cyclic adenosine monophosphate (cAMP). In addition, CBD also acts as an inverse agonist of the GPCRs, such as GPR3, GPR6 and GPR12, which are constitutively active and present in neurons, and as a partial agonist of peroxisome proliferator-activated receptor-gamma (PPARγ), serotonin 5-HT1A receptor and transient receptor potential cation channel subfamily V member 1 (TRPVA1) [13].

Our research team has already demonstrated in vitro that CBD promotes human gingiva-derived MSCs (hGMSCs) towards neuronal progenitor cells (NPCs) differentiation and neurogenesis, highlighting the effects of CBD on differentiation [14]. In the present study, we decided to use a different cell lineage, the spinal cord × neuroblastoma hybrid cell line (NSC-34). These cells are immature motoneurons in a proliferative state [15] that do not express CB2, which is coherent with the fact that this receptor type is preferentially expressed in glial elements [16]. The lack of CB2 allows us to focus on the CB1-activated signaling cascade: in this context, the aim of our work is to investigate how CBD changes the expression of genes associated with neuronal differentiation. Moreover, thanks to the important difference in the action profile of CBD, we treated the cells with two concentrations of this phytocompound in order to understand its effect on neuronal differentiation. 

## 2. Results

### 2.1. Transcriptomic Analysis

The analysis of the comparison between NSC-34 (CTRL) and NSC-34 treated with the CBD at 5 µM concentration (CTRL vs. 5 µM) shows 5098 differentially expressed genes (DEGs), while NSC-34 (CTRL) and NSC-34 treated with the CBD at 10 µM concentration (CTRL vs. 10 µM) shows 5909 DEG.

**Figure 2:**
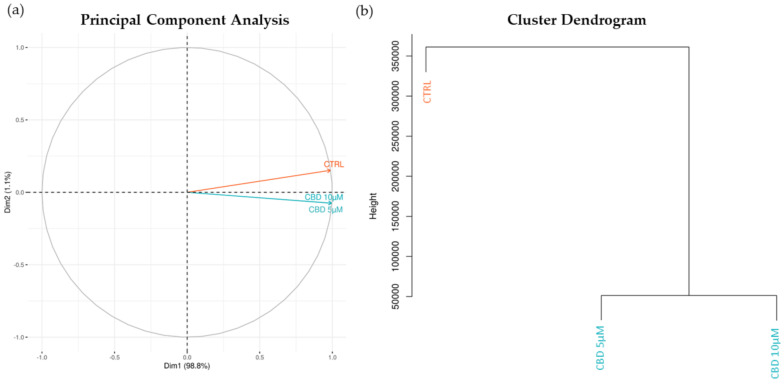
Principal Component Analysis (PCA) (**a**) and Cluster Dendrogram (**b**) of the CTRL and CBD groups obtained from the transcriptomic profile. In orange, we can see the CTRL group, whereas in blue, CBD 5 µM and CBD 10 µM are represented. Both the PCA and the dendrogram show that the two groups treated with CBD are both very distant from the CTRL group.

We firstly performed the clustering analysis of the CTRL, CBD 5 µM and CBD 10 µM, as shown in Figure 2. Both the Principal Component Analysis in panel a and the hierarchical clustering dendrogram in panel b highlight how dissimilar the CTRL group is compared to the treatments. Additionally, both for CTRL vs. 5 µM (Table S1) and CTRL vs. 10 µM (Table S2), we performed the Gene Set Enrichment analysis. The Gene Ontology inspection revealed 2974 and 3157 enriched biological processes, respectively.

Transcriptomic analysis shows that some of the genes encoding the receptors in which CBD binds are significantly expressed. Among these genes, we are particularly interested in the upregulation of *Cnr1, Gpr3* and *Gpr6,* which, indeed, are GPCRs genes. Genes that encode for other receptors such as PPARγ, 5-HT1A and TRPVA1 are not differentially expressed. In Table 1, we studied DEGs at different concentrations of CBD, in order to evaluate its binding to GPCRs. *Cnr1* expression is increased at both concentrations, the genes associated with the other G protein receptors show some differences: the gene *Gpr3*, which encodes for GPR3, shows a decreased expression at 10 µM, while the gene *Gpr6*, which encodes for GPR6, is upregulated at 5 µM. We also studied *Gnai,* which encodes for G Protein Subunits Alpha, *Gnas*, which encodes for the stimulatory G-protein subunit α, *Grk*, which encodes for G protein-coupled receptor kinases, and *Arrb*, which encodes for β-arrestins. *Arrb* and *Grk* encode for fundamental proteins for GPCRs desensitization and could also activate specific pathways. 

In Table 2, the DEGs of the subunit of protein kinase A (PKA), a key protein of the cAMP/PKA pathway, are reported. The activation of this protein depends on cAMP, so we decide to study the main protein because CBD lower the levels of cAMP. *Prkaca* is the gene that encodes one of the catalytic subunits, while the others are regulatory subunits that inhibit the activation of the PKA.

In Table 3 are reported the DEGs that encode for extracellular signal-regulated kinases (ERK), key proteins of the MAPK cascade that is activated by the G-proteins. ERK can induce neurodifferentiation phosphorylating cAMP response element-binding proteins (CREBs). *Atf4* is a gene that encode for CREB2. *Rps6ka3* is a gene that encodes for RSK2, a protein that activates Crebs proteins using p-ERK.

Table 4 shows DEGs of minichromosome maintenance protein complex (MCM) and the genes associated with the proliferation, in which the activation of MAPK is also involved. For this reason, we study the DEGs markers of proliferations, such as *Cdc6, Pcna* and *Mki67*, which are downregulated. The MCM proteins are fundamental for DNA replication and an early marker of proliferations.

In Table 5 are reported the DEGs involved in PI3K/AKT pathway. PI3K/AKT is a pathway that could be activated after interaction between CB1 and CBD, and is involved in neuronal differentiation. In particular, *Akt3* is highly expressed in brain cells.

**Figure 3:**
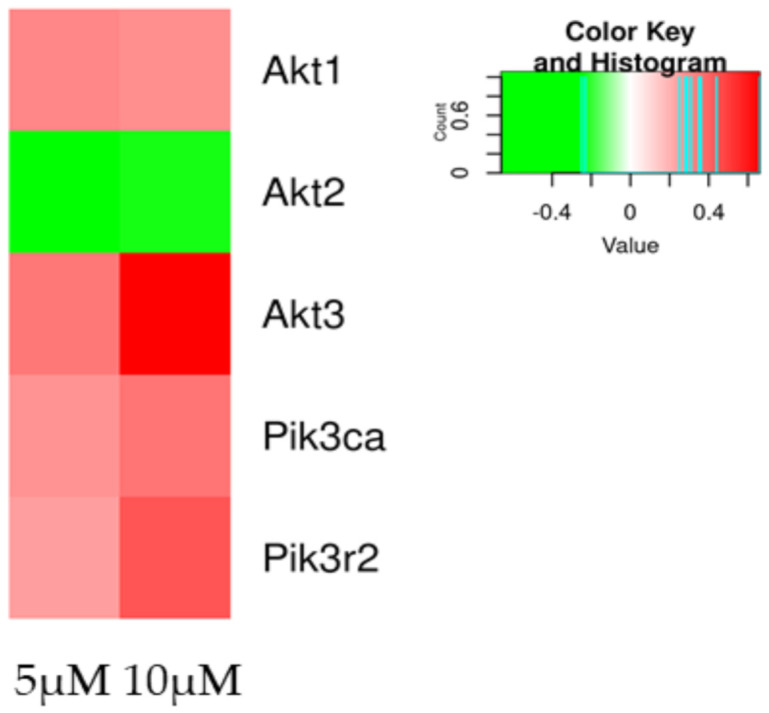
Heatmap of DEGs involved in the DEGs involved in the PI3K/AKT pathway genes in the comparison of CTRL vs. 5 µM (left column of each comparison) or CTRL vs. 10 µM (right column of each comparison). The green scale is related to downregulated genes, whereas the red palette represents upregulated ones. Gray is used when the difference is not statistically relevant in the comparison.

Figure 3 shows a heatmap of DEGs involved in the PI3k/Akt at both concentrations. DEGs show a similar trend, except for *Akt3*, which is increased at 10 µM.

In Table 6 are reported the DEGs involved in the Wnt pathway, which is associated with the neurodifferentiation. CB1 activates AKT by phosphorylation. p-AKT can inhibit Glycogen synthase kinase-3 beta (GSK-3β), a key protein of the Wnt pathway.

In Table 7, we study reported DEGs used, such as markers of neurodifferentiation. As shown, 5 µM of CBD are able to upregulate only a few markers of neuronal differentiation compared to 10 µM, suggesting that this higher concentration of CBD is more efficient than 5 µM in inducing differentiation in the NSC-34 cell line.

In Table 8, DEGs associated both with circadian regulation and neurodifferentiation are shown. *Hspa1b* is a gene that encode for a shuttle of cannabinoids inside the cells. *Rorb* encodes for Retinoic Acid Receptor-Related Orphan beta. This gene, overexpressed only at 10 µM, is associated with neuron differentiation.

**Figure 4:**
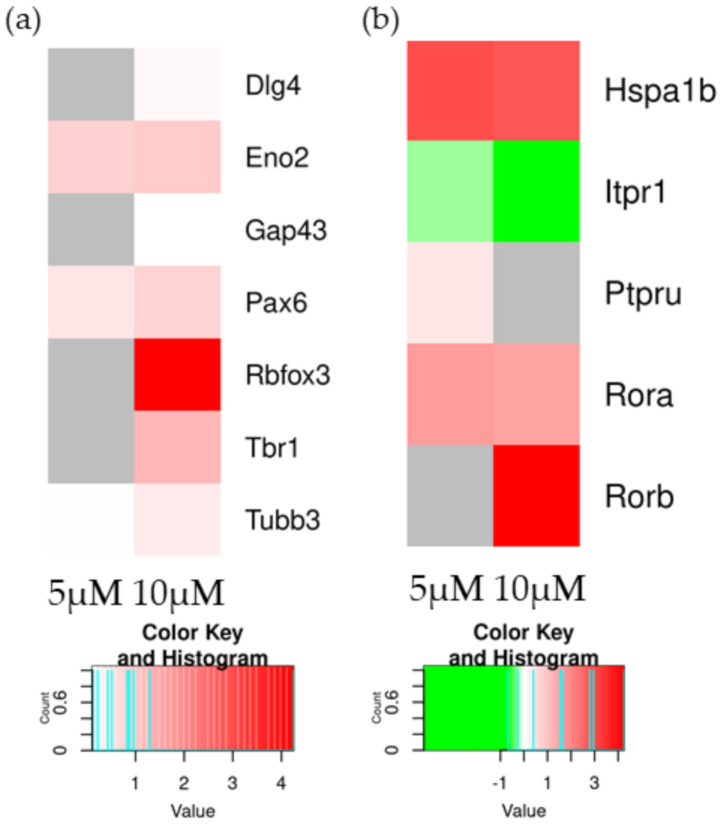
Heatmap of DEGs associated with neurodifferentiation (**a**) and genes involved in the activations of RORs (**b**) in the comparison of CTRL vs. 5 µM (left column of each comparison) or CTRL vs. 10 µM (right column of each comparison). The green scale is related to downregulated genes, whereas the red palette represents upregulated ones. Gray is used when the difference is not statistically relevant in the comparison.

In Figure 4a, a heatmap of DEGs associated with neurodifferentiations is shown; we clearly see an upregulation of different genes at 10 µM, such as *Rbfox3,* at 5 µM when fewer genes are present. In Figure 4b, DEGs are involved in the neuronal differentiation with the RORs. *Rorb* a gene involved in neuronal differentiation is upregulated at 10 µM, while *Rora* is upregulated at both concentrations.

### 2.2. Western Blot Analyses

**Figure 5:**
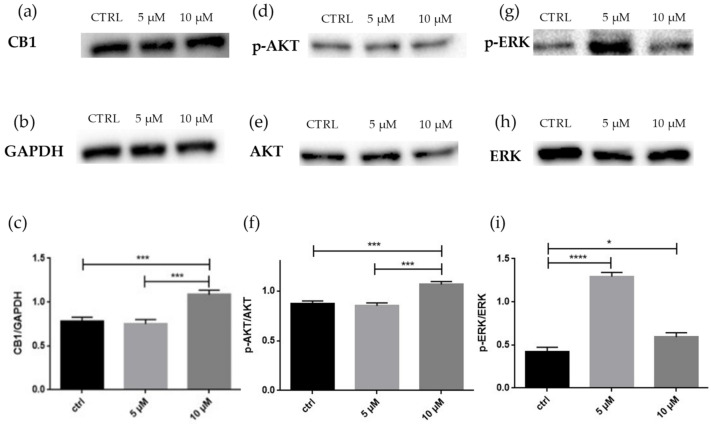
(**a**) Evidence of the significant increase of CB1 in NSC-34 after treatment with CBD at a high dose. (**b**) GAPDH used for normalization. (**c**) Densitometric analysis of CB1, *** *p* < 0.0005. (**d**) Evidence of phosphorylation of ATK at a high dose. (**e**) Non-phosphorylated AKT used for normalization. (**f**) Densitometric analysis of p-AKT, *** *p* < 0.0005. (**g**) Evidence of different phosphorylation of ERK at two doses compared to the control. (**h**) Non-phosphorylated ERK used for normalization. (**i**) Densitometric analysis of p-ERK, * *p* < 0.05, **** *p* < 0.0001.

In order to confirm transcriptomic data, Western blot assays for selected proteins were performed. As seen in Figure 5, the statistical analysis revealed that CB1 levels significantly increased at 10 μM, while there was no upregulation of this receptor at 5 μM. The phosphorylation of AKT was present only at 10 μM. The phosphorylation of ERK1/2 increased at 5 μM but strongly decreased at 10 μM, although it remained higher than the control.

## 3. Discussion

The increase of CB1 expression and its signal transduction pathways have been already associated with neuronal differentiation [4,5,17,18]. The overexpression of CB1 in the NSC-34 is associated with the neurodifferentiation [16]. In this work, we wanted to evaluate if 5 µM or 10 µM of CBD, one of the main cannabinoids, can induce neurodifferentiation triggering the signaling cascade of CB1. Our transcriptomic analysis shows, see Figure 2a,b, that treatment with CBD can exert an effect on our cellular models.

Western blot analysis in line with transcriptomic data suggests that 10 µM of CBD can efficiently trigger CB1 production (Figure 5c).

CB1 signaling activates the MAPKs, PI3K/AKT and mammalian target of rapamycin (mTOR) pathways, while the activation of Gi proteins mediates the inhibition of adenylyl cyclase (AC) and PKA [19]. The binding of CBD to CB1, GPR3, GPR6 and GPR12 leads to decreasing level of cAMP [20]. In our study (Table 1), the gene *Gnas,* which encodes for the stimulatory G-protein subunit α, is slightly upregulated at 10 µM, while it is downregulated at 5 µM. On the other hand, there was an increased expression of the genes associated to Gi proteins at both concentrations. This evidence, in accordance with the literature, leads to the hypothesis that cAMP is decreased. Indeed, the inhibition of cAMP/PKA pathway is also suggested by the upregulation of genes encoding for the regulatory protein of PKA, the effect of which is inhibitory. At 5 µM, the genes *Prkar1b*, *Prkar2a* and *Prkar2b* are upregulated, while at 10 µM, *Prkar1a*, *Prkar2a* and *Prkar2b* are also upregulated, as well as *Prkaca,* which encodes for the catalytic subunit Prkaca of PKA (Table 2). 

The GPCRs family also mediated their effect thanks to the G protein-coupled receptor kinases (GRKs) and the arrestins. These classes of proteins not only have the function of destabilizing and internalizing the receptor, but also to participate in the signaling cascade. Herein, *Arrb1*, which encodes for β-arrestin 1, was overexpressed at both concentrations, while *Arrb2*, which encodes for β-arrestin 2, was increased at 5 µM and does not change significantly at 10 µM. Different GRKs were upregulated; these proteins are fundamental for the desensitization of the G-proteins, as they contribute to increase the receptor affinity for the β-arrestins. This means that the cell was reacting by desensitizing the active GPCRs and, consequently, activating specific pathways. In particular, β-arrestin 1 stimulates ERK 1/2, SRC proto-oncogene non receptor tyrosine kinase (Src) and Mitogen-activated protein kinase kinase 1/2 (MEK 1/2) phosphorylation, while β-arrestin 2 is fundamental for the internalization of the receptor but also activate ERK1/2 and Src [21]. According to the literature, 2-AG, an agonist of CB1, can induce prolonged phosphorylation of ERK1/2, c-Jun N-terminal kinases (JNK) ½/3, CREB, and p38 mitogen-activated protein kinases via β-arrestin 1 within 5 minutes after ligand incubation [22].

A study demonstrated that CB1 agonist activation promotes neuronal differentiation and maturation of neural stem cells (NSCs). The results, obtained 1, 2 and 3 days after treatments, showed that the levels of ERK1/2 phosphorylation decline during NSCs differentiation [23]. In contrast, the activation of the ERK1/2 signaling pathway is proved fundamental for the neurite outgrowth and the correct neurogenesis [24]. Indeed, the Western blot analysis confirmed an increased amount of p-ERK in treated cells compared to the control (Figure 5i). The phosphorylation of ERK, as mentioned above, can also be given by the activation of β-arrestin: since CBD binds to the allosteric site of CB1, the receptor is destabilized, and this results in the activation of β-arrestin, which, in turn, increase the phosphorylation of ERK [8]. Moreover, the increased expression of *Gpr6* at 5 µM may influence the increase of p-ERK [25], while at 10 µm, the lower level of p-ERK compared to 5 µM can be justified by the downregulation of *Gpr3* [20,26]. p-ERK can activate CREBs through Ribosomal S6 Kinase 1 (RSK1) and Ribosomal S6 Kinase 2 (RSK2). CREBs are important transcriptional factors for the early stages of cortical neuron differentiation [27]. Treatment with retinoic acid (RA) to induce neurodifferentiation show early stimulation of ERK1/2, which is required for CREBs phosphorylation and transcriptional activity [28]. The transcriptomics analysis showed the upregulation at both concentration of the gene *Rps6ka3* that encode for RSK2, that is higher at 10 µM. An important difference between 5 µM and 10 µM are the genes that encode for CREBs: at 5 µM, there is a downregulation of the gene *Creb1*, while at 10 µM, there is an upregulation of *Atf4,* which encodes for Creb2, but there is also a downregulation of *Creb5*. This evidence suggests that 10 µM of CBD, through the phosphorylation of ERK and consequently the phosphorylation of RSK2, is able to activate CREBs in order to induce neuronal differentiation (Table 3).

The phosphorylation of ERK1/2 is associated with the mitosis activation. In order to exclude ongoing active replication, the Kegg enrichment for “DNA replication” was inspected. *Cdc6,* which encodes for the protein cell division cycle 6, an essential regulator for the DNA replication, was highly downregulated at both doses, and this, as a consequence, downregulates the gene encoding for MCM. *Mki67,* which encodes for the proliferation marker protein Ki-67, was downregulated at both concentrations, confirming a block of the DNA replication and consequently of the mitosis [29]. The gene *Pcna* that encode for the protein proliferating cell nuclear antigen (PCNA) is downregulated at both concentrations. This protein is associated with the proliferations as fundamental for DNA replication [30,31] (Table 4). 

One pathway associated with differentiation after CB1 activation is the PI3K/AKT pathway. The transcriptomics analysis in Figure 3 shows both at 5 µM and 10 µM an upregulation of the genes of PI3K and AKT (Table 5). After performing the Western blot, shown in Figure 5f, the phosphorylation of AKT was present only at 10 µM, suggesting the effect was mediated by CB1 at 10 µM. AKT is involved in many vital cell processes, such as proliferation and differentiation, and studies correlate the presence of p-AKT as necessary to achieve differentiation, also in retinoic-acid induced differentiation [23,32]. 

P-AKT inhibits GSK-3β by phosphorylation. The link between these proteins represents a link between the two pathways of AKT and the Wnt one. The activation of Wnt is associated with neurodifferentiation [33,34].

If GSK-3β is not phosphorylated, it inhibits β-catenin. At 5 µM, there is no p-AKT and the gene that encodes for GSK-3β is upregulated, but we see a decrease of the gene *Ctnnb1*, which encodes for the protein β-catenin, and also the gene *Axin*2, which encode for Axin2, a fundamental protein for the stability of the β-catenin. At 10 µM *Ctnnb1* show no change and p-AKT can inhibit GSK-3β, this means that β-catenin can bind its transcription factors, which are the T-cell factor/lymphoid enhancer-binding factor (TCF/LEF) family. At 10 µM, there is a minor increase of *Lef1*, which encodes for one of the proteins that composed the transcription factor, but there is also an increased expression of different genes associated with the inhibitor of β-catenin, like *Chd8*, *Ctbp2* and *Tle3.* Indeed, this pathway has been shown to induce the expression of *Neurod1*, which in turn promotes cell cycle exit and neuronal differentiation [33]. At 10 µM, the gene *Neurod1* is downregulated. At 10 µM, there is a downregulation of *Neurod1* and the upregulation of the gene that encode for the inhibitory protein of this pathway. This suggests that the neurodifferentiation associated with p-AKT and CBD is not correlated with Wnt signaling (Table 6).

At 10 µM, the increase of p-ERK and p-AKT is correlated with the absence of DNA replication and proliferation, which suggests the beginning of differentiation.

In order to confirm if neuronal differentiation is taking place, the genes involved in these processes were investigated. In Figure 4a, we can see a great expression of *Rbfox3* at 10 µM but not at 5 µM. *Rbfox3* encodes for the protein NeuN, which is required to promote the neuronal differentiation of postmitotic neurons, and for this reason, it is the most used marker [35]. The gene *Gap43* is expressed at high levels in neuronal growth cones during development [36]. This gene is slightly overexpressed at 10 µM. Postsynaptic density protein 95 (PSD95) is involved in the maturation in spine hippocampal neurons in vivo [37]. In our study, the gene *Dlg4,* which encodes for PSD95, is upregulated only at 10 µM. There are also some genes associated with the neurodifferentiation upregulated both in 5 µM and 10 µM, such as *Pax6, Tubb3* and *Eno2* (Table 7) [38,39]. Paired Box 6 (Pax6) and T-Box Brain Transcription Factor 1/2 (Tbr1/2) are transcriptional factors fundamental for the creation of a correct epigenetic environment for the neurodifferentiation. The gene that encodes for Tbr1 is upregulated only at 10 µM.

Using the transcriptomics analysis, we study the correlation between the treatment of CBD the retinoic acid receptor-related orphan receptors (RORs). RORs are involved in the circadian rhythm [40], but also in neuronal differentiation [41,42]. Given the experimental nature of our work, it is difficult to think of a conventional activation of the circadian cycle, so we can say that ROR is used for neurodifferentiation.

In order to understand how CBD could reach nuclear receptors, it is important to mention that endocannabinoids, such as N-arachidonoylethanolamine (AEA), or cannabinoids, such as THC, are capable of traversing the plasma membrane bilayer unaided thanks to their chemical nature. CBD is proved to bind nuclear receptors such as PPAR-γ. The lipophilicity and the ability of a substance to cross the lipid membrane is expressed by the logarithm of the partition coefficient in n-octanol/water (CLogP). CBD, THC and AEA are all uncharged lipids [43] and have CLogP values of 6.64, 7.23 and 5.1, respectively [44,45]. The uptake of AEA occurs by passive diffusion, facilitated diffusion and/or endocytosis [46]. Inside the cell, Heat Shock Protein 70 kilodaltons (Hsp70), encoded by the gene *Hspa1b*, may act as an “AEA chaperon” at the plasma membrane, where it might promote the desorption of this lipid, thus facilitating its transport through the cytoplasm, since AEA and CBD are both non-polar lipid molecules and bind to similar proteins [47]. In our study, there is an increase of the gene *Hspa1b* at both concentrations. We can speculate that CBD can enter inside the cell also by diffusion and be transported in a similar way to AEA. Inside the cell, CBD, linked to Hsp70, can be delivered to the endoplasmic reticulum or in the nucleus for transcriptional regulation, interacting with different receptors, such as PPAR-γ.

The three RORs, α, β and γ, are transcriptional regulator binding-specific DNA sequence, known as the ROR response element (RORE). RORs have different functions inside the cells, from the regulation of metabolism to the regulation of the immune system [40]. According to the aim of our study, we analyzed only the function related to neurodifferentiation.

RORβ mediates several neurodevelopmental functions. A total deletion of RORβ in mice causes sleep disorder, hindlimb motor control, opsin induction in cone photoreceptors and differentiation of rod photoreceptors [48]. At a cellular level, it controls key steps in transcriptional pathways that direct the differentiation of neuronal cell types. Moreover, in immature neurons, RORβ influences the cell fate outcome in several differentiation pathways [42,49]. In our results, the gene *Rorb* was highly overexpressed at 10 µM, suggesting a correlation between the use of CBD, RORβ and neurodifferentiation.

**Figure 6:**
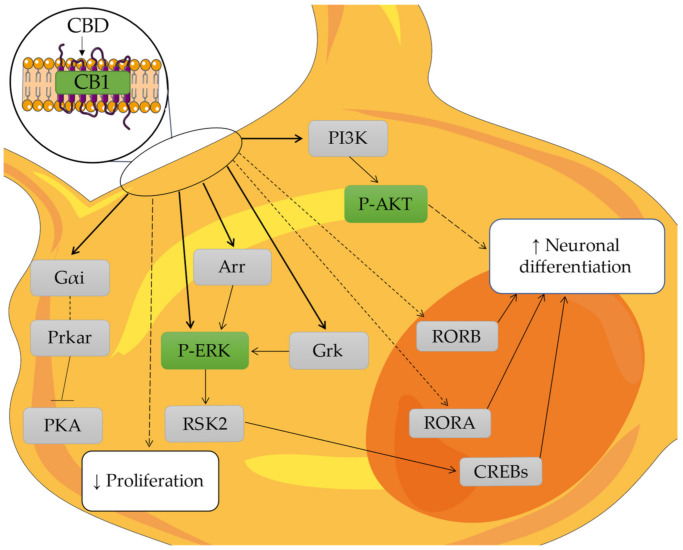
Proposed mechanism for the neurodifferentiation of NSC-34 with the CB1 signaling at 10 µM CBD. The image shows the change inside the NSC-34. In green, the protein analyzed with WB is shown; in light gray, the proteins encoded by the genes analyzed with the transcriptomics analyses are shown; and in the white square, the biological process is shown. Continuous arrows indicate direct associations, while dashed arrows indicate indirect associations. Figure drawn using the vector image bank of Servier Medical Art by Servier (http://smart.servier.com/, accessed on 23 July 2022). Licensed under a Creative Commons Attribution 3.0 Unported License (https://creativecommons.org/licenses/by/3.0/, accessed on 23 July 2022).

RORα plays a key role in the regulation of circadian rhythm, but also controls the Purkinje cell maturation and differentiation through transcriptional gene, such as *Shh, Slc1a6, Itpr1, Ptpru* and *Pcp4* [50,51,52], which are not differentially expressed in our experimental set, apart from the downregulation of *Itpr1* at both concentration the proteins. This could be caused by the short exposure time and that *Itpr1* is correlated with other neuronal functions. In our study, the gene that encode for RORα, *Rora*, is upregulated at both concentrations (Table 8). In Figure 6, our hypothesized mechanism is shown for the neuronal differentiations of NSC-34 with the concentration of 10 µM CBD.

## 4. Materials and Methods

### 4.1. CBD Extraction from Cannabis Sativa

CREA-CIN (Rovigo, Italy) was the provider of Cannabis Sativa, according to the authorization SP/106 23/05/2013 of the Ministry of Health (Rome, Italy). 

CBD, at a purity higher than 99%, was isolated from Cannabis Sativa using the standardized protocol, which avoids the presence of THC [53,54].

### 4.2. NSC-34 Culture and Treatment

NSC-34 cell line were provided by Cedarlane Corporation (Burlington, ON, Canada) and it was maintained in DMEM High Glucose, 10% Fetal Bovine Serum, 1% penicillin/streptomycin and 1% L-Glutamine. All reagents were provided by Sigma-Aldrich, Merck KGaA (Darmstadt, Germany). The incubator for the cells was set at 37 °C with 5% of CO_2_.

In order to obtain the material for transcriptomic analysis and Western blot, cells were seeded in 6-well plates with a density of 300,000 cells/cm^2^. The culture medium was then replaced with different concentrations of CBD (5 µM and 10 µM in DMSO < 0.1%) and allowed to exert its effect for 24 hours. In order to have a valid control, control wells underwent a complete medium change. After 24 hours, cells were harvested for further analyses.

### 4.3. Library Preparation and Bioinformatics Inspection 

After centrifugation and supernatant discard, the pellet used for transcriptomic analysis underwent RNA extraction reported in the manufacturer instructions of the Maxwell^®^ RSC simplyRNA Cells Kit (Promega, Madison, WI, USA). TruSeq RNA Exome protocol (Illumina, San Diego, CA, USA) was followed for the preparation of the library, as previously reported [55]. Illumina instrument MiSeq was then used to analyze the library.

The quality of the CTRL, CBD 5 µM and CBD 10 µM raw data was inspected with fastqc 0.11.4 (Babraham Institute, Cambridge, UK). All the bases with low score were dropped using Trimmomatic 0.40 (Usadel Lab, Aachen, Germany) [56]. Additionally, the adapters were removed in the same way. Then, alignment and counting of the transcript were performed with Spliced Transcripts Alignment to a Reference (STAR) RNA-seq aligner 2.7.10a (New York, NY, USA) [57] and the python package htseq-count 0.13.5 (European Molecular Biology Laboratory (EMBL), Heidelberg, Germany) [58], respectively, against the mouse reference genome GRCm39 with primary transcript assembly M28. The analysis of DEGs for CTRL against 5 µM or CTRL against 10 µM groups were done with DESeq2 library [59] on R 4.2.0 (R Core Team). The post-hoc Benjamini–Hochberg procedure was chosen to remove the false positive DEGs using a threshold of 0.05. For this reason, we did not apply any fold-change cutoff.

### 4.4. Western Blot Analyses

After centrifugation and supernatant discard, the proteins were extracted from the pellet using RIPA Buffer and quantified using Bradford Assay (Bio-Rad, Hercules, CA, USA). After denaturation at 95 °C, 25 µg of protein for each samples underwent separation using SDS-polyacrylamide gel electrophoresis (SDS-PAGE) and then they were transferred on PVDF membrane (Immobilon–P, Millipore, Burlington, MA, USA). Then, 5% skimmed milk in TBS was used for blocking at room temperature for 1 hour, followed by the overnight incubation at 4 °C. The antibodies used were: Anti-CB1 (1:500, ThermoFisher Scientific, Rockford, IL, USA), anti-GAPDH HRP conjugate (1:1000, Cell Signaling, Danvers, MA, USA), anti-p-Akt (1:1000; Cell Signaling, Danvers, MA, USA), anti-Akt (1:1000, Cell Signaling, Danvers, MA, USA), anti-p-ERK1/2 (1:2000; Cell Signaling, Danvers, MA, USA) and anti-ERK2 (1:1000; Cell Signaling, Danvers, MA, USA). The antibody mouse anti-rabbit IgG-HRP (1:2000, Santa Cruz Biotechnology, Dallas, TX, USA) was used as a secondary antibody to be incubated with the membranes for 1 h at room temperature. In order to acquire the bands, ChemiDoc™ MP System (Bio-Rad) was after exposition to enhanced chemiluminescence system (Luminata Western HRP Substrates, Millipore, Burlington, MA, USA).

## 5. Conclusions

Treatment with CBD, after 24 hours of administration in NSC-34 cells, proved to be able to trigger neuronal differentiation markers. In particular, 10 µM of CBD seemed to be the dose which could better trigger these signals. The pathway starts at CB1, then phosphorylation of ERK1/2, followed by the activation of AKT. Transcriptomic data and Western blot analysis highlight 10 µM of CBD as the dose where AKT is phosphorylated; indeed, the pathway of MAPKs and PI3K/AKT are both important for the neuronal differentiation. Additionally, 10 µM of CBD is the dose where more genes associated with the neurodifferentiation are present compared to 5 µM. According to the literature, RORs have already been associated to neurodifferentiation; however, using the transcriptomic analysis, we provided, for the first time to our knowledge, a new correlation between CBD and RORs.

## Figures and Tables

**Table 1:** DEGs associated with the G protein-coupled receptors and CBD.

Genes	CTRL vs. 5 µM Fold Change	CTRL vs. 5 µM *q*-Value	CTRL vs. 10 µM Fold Change	CTRL vs. 10 µM *q*-Value
*Cnr1*	0.47	3.97 × 10^−8^	0.70	1.03 × 10^−17^
*Gpr3*	-	>0.05	−1.18	5.95 × 10^−4^
*Gpr6*	0.89	1.05 × 10^−2^	-	>0.05
*Gnai1*	1.45	5.75 × 10^−4^	-	>0.05
*Gnai2*	0.17	2.46 × 10^−7^	0.46	5.42 × 10^−54^
*Gnai3*	-	>0.05	0.29	3.55 × 10^−10^
*Gnas*	−0.22	1.11 × 10^−98^	0.08	7.73 × 10^−16^
*Grk2*	-	>0.05	−0.17	9.88 × 10^−4^
*Grk5*	-	>0.05	0.31	4.50 × 10^−2^
*Grk6*	0.49	6.17 × 10^−9^	0.39	6.81 × 10^−6^
*Arrb1*	0.36	1.78 × 10^−3^	0.54	5.94 × 10^−7^
*Arrb2*	0.14	2.22 × 10^−2^	-	>0.05

The fold change for the analysis of CTRL vs. 5 µM was computed as log_2_ (5 µM/CTRL). The fold change for the analysis of CTRL vs. 10 µM was computed as log_2_ (10 µM/CTRL). The hyphen symbol in the fold change column is used when *q*-value > 0.05. All fold changes are rounded to the second decimal digit.

**Table 2:** DEGs of the subunits of the PKA protein.

Genes	CTRL vs. 5 µM Fold Change	CTRL vs. 5 µM *q*-Value	CTRL vs. 10 µM Fold Change	CTRL vs. 10 µM *q*-Value
*Prkaca*	-	>0.05	0.50	3.88 × 10^−26^
*Prkar1a*	-	>0.05	0.42	1.85 × 10^−72^
*Prkar1b*	0.83	3.67 × 10^−2^	-	>0.05
*Prkar2a*	0.29	8.07 × 10^−4^	0.59	1.50 × 10^−13^
*Prkar2b*	0.27	1.74 × 10^−3^	0.34	5.47 × 10^−5^

The fold change for the analysis of CTRL vs. 5 µM was computed as log_2_ (5 µM/CTRL). The fold change for the analysis of CTRL vs. 10 µM was computed as log_2_ (10 µM/CTRL). The hyphen symbol in the fold change column is used when *q*-value > 0.05. All fold changes are rounded to the second decimal digit.

**Table 3:** DEGs of ERK and activation of Creb.

Genes	CTRL vs. 5 µM Fold Change	CTRL vs. 5 µM *q*-Value	CTRL vs. 10 µM Fold Change	CTRL vs. 10 µM *q*-Value
*Mapk3*	0.32	1.23 × 10^−11^	0.67	1.61 × 10^−52^
*Mapk1*	0.23	2.34 × 10^−6^	0.58	4.72 × 10^−38^
*Rps6ka3*	0.11	3.68 × 10^−3^	0.54	9.21 × 10^−66^
*Creb1*	−0.17	4.50 × 10^−2^	-	>0.05
*Atf4*	-	>0.05	0.28	4.67 × 10^−23^
*Creb5*	-	>0.05	−1.2	8.90 × 10^−3^

The fold change for the analysis of CTRL vs. 5 µM was computed as log_2_ (5 µM/CTRL). The fold change for the analysis of CTRL vs. 10 µM was computed as log_2_ (10 µM/CTRL). The hyphen symbol in the fold change column is used when *q*-value > 0.05. All fold changes are rounded to the second decimal digit.

**Table 4:** DEGs of MCM and the genes associated with the proliferation.

Genes	CTRL vs. 5 µM Fold Change	CTRL vs. 5 µM *q*-Value	CTRL vs. 10 µM Fold Change	CTRL vs. 10 µM *q*-Value
*Cdc6*	−2.53	6.14 × 10^−5^	−2.10	2.41 × 10^−4^
*Mcm3*	−0.15	1.17 × 10^−3^	−0.13	3.78 × 10^−3^
*Mcm5*	−0.41	4.26 × 10^−8^	−0.72	2.43 × 10^−20^
*Mcm6*	−0.25	3.67 × 10^−5^	−0.17	6.11 × 10^−3^
*Mcm9*	-	>0.05	0.39	3.02 × 10^−2^
*Mcm10*	−0.35	3.32 × 10^−2^	−0.56	5.43 × 10^−4^
*Pcna*	−0.47	1.02 × 10^−2^	−0.65	3.40 × 10^−4^
*Mki67*	−0.49	2.92 × 10^−8^	−0.40	5.75 × 10^−6^

The fold change for the analysis of CTRL vs. 5 µM was computed as log_2_ (5 µM/CTRL). The fold change for the analysis of CTRL vs. 10 µM was computed as log_2_ (10 µM/CTRL). The hyphen symbol in the fold change column is used when *q*-value > 0.05. All fold changes are rounded to the second decimal digit.

**Table 5:** DEGs of MCM and the genes involved in the PI3K/AKT pathway.

Genes	CTRL vs. 5 µM Fold Change	CTRL vs. 5 µM *q*-Value	CTRL vs. 10 µM Fold Change	CTRL vs. 10 µM *q*-Value
*Akt1*	0.31	3.45 × 10^−23^	0.29	2.72 × 10^−20^
*Akt2*	−0.23	5.43 × 10^−5^	−0.25	0.000172
*Akt3*	0.35	3.51 × 10^−34^	0.66	4.74 × 10^−131^
*Pik3ca*	0.28	0.00229	0.36	5.13 × 10^−5^
*Pik3r2*	0.25	4.88 × 10^−7^	0.44	5.73 × 10^−21^

The fold change for the analysis of CTRL vs. 5 µM was computed as log_2_ (5 µM/CTRL). The fold change for the analysis of CTRL vs. 10 µM was computed as log_2_ (10 µM/CTRL). The hyphen symbol in the fold change column is used when *q*-value > 0.05. All fold changes are rounded to the second decimal digit.

**Table 6:** DEGs of the Wnt pathway involved in the activation of β-catenin.

Genes	CTRL vs. 5 µM Fold Change	CTRL vs. 5 µM *q*-Value	CTRL vs. 10 µM Fold Change	CTRL vs. 10 µM *q*-Value
*Ctnnb1*	−0.22	5.50 × 10^−16^	−	>0.05
*Lef1*	0.51	3.70 × 10^−14^	0.75	3.12 × 10^−31^
*Chd8*	-	>0.05	0.19	9.56 × 10^−10^
*Ctbp2*	0.14	1.54 × 10^−2^	0.35	1.41 × 10^−11^
*Tle3*	0.35	2.22 × 10^−23^	0.51	2.26 × 10^−51^
*Neurod1*	-	>0.05	−0.52	9.96 × 10^−3^

The fold change for the analysis of CTRL vs. 5 µM was computed as log_2_ (5 µM/CTRL). The fold change for the analysis of CTRL vs. 10 µM was computed as log_2_ (10 µM/CTRL). The hyphen symbol in the fold change column is used when *q*-value > 0.05. All fold changes are rounded to the second decimal digit.

**Table 7:** DEGs involved in neurodifferentiation.

Genes	CTRL vs. 5 µM Fold Change	CTRL vs. 5 µM *q*-Value	CTRL vs. 10 µM Fold Change	CTRL vs. 10 µM *q*-Value
*Rbfox3*	-	>0.05	4.25	1.17 × 10^−2^
*Tubb3*	0.12	1.23 × 10^−44^	0.43	0
*Gap43*	-	>0.05	0.11	2.06 × 10^−2^
*Pax6*	0.51	7.14 × 10^−14^	0.83	2.64 × 10^−38^
*Dlg4*	-	>0.05	0.22	1.41 × 10^−3^
*Tbr1*	-	>0.05	1.29	4.94 × 10^−2^
*Eno2*	0.87	1.57 × 10^−13^	0.96	1.94 × 10^−16^

The fold change for the analysis of CTRL vs. 5 µM was computed as log_2_ (5 µM/CTRL). The fold change for the analysis of CTRL vs. 10 µM was computed as log_2_ (10 µM/CTRL). The hyphen symbol in the fold change column is used when *q*-value > 0.05. All fold changes are rounded to the second decimal digit.

**Table 8:** DEGs associated with RORs.

Genes	CTRL vs. 5 µM Fold Change	CTRL vs. 5 µM *q*-Value	CTRL vs. 10 µM Fold Change	CTRL vs. 10 µM *q*-Value
*Hspa1b*	2.96	1.50 × 10^−02^	2.80	1.91 × 10^−02^
*Rorb*	-	>0.05	4.25	1.17 × 10^−02^
*Itpr1*	−0.27	3.52 × 10^−02^	−0.68	5.51 × 10^−08^
*Ptpru*	0.40	3.77 × 10^−03^	−	>0.05
*Rora*	1.65	1.34 × 10^−03^	1.54	2.53 × 10^−03^

The fold change for the analysis of CTRL vs. 5 µM was computed as log_2_ (5 µM/CTRL). The fold change for the analysis of CTRL vs. 10 µM was computed as log_2_ (10 µM/CTRL). The hyphen symbol in the fold change column is used when *q*-value > 0.05. All fold changes are rounded to the second decimal digit.

## Data Availability

The data presented in this study are openly available in the NCBI Sequence Read Archive at BioProject accession numbers PRJNA860777, PRJNA839187 and PRJNA719259.

## Supplementary Materials

The following supporting information can be downloaded at: https://www.mdpi.com/article/10.3390/molecules27175644/s1. Table S1: Gene Set Enrichment analysis for CTRL vs. 5 µM; Table S2: Gene Set Enrichment analysis for CTRL vs. 10 µM.

## Sample Availability

Samples of the compounds CBD and NSC-34 cellsare available from the authors.

## References

[1] Crocq M.-A. (2020). History of cannabis and the endocannabinoid system. Dialogues Clin. Neurosci..

[2] Cristino L., Bisogno T., Di Marzo V. (2020). Cannabinoids and the expanded endocannabinoid system in neurological disorders. Nat. Rev. Neurol..

[3] Oddi S., Scipioni L., Maccarrone M. (2020). Endocannabinoid system and adult neurogenesis: A focused review. Curr. Opin. Pharmacol..

[4] Maccarrone M., Guzmán M., Mackie K., Doherty P., Harkany T. (2014). Programming of neural cells by (endo)cannabinoids: From physiological rules to emerging therapies. Nat. Rev. Neurosci..

[5] Zorina Y., Iyengar R., Bromberg K.D., Bradshaw R.A., Dennis E.A. (2010). Chapter 203-Effectors of Gα_o_. Handbook of Cell Signaling.

[6] Pertwee R.G., Howlett A.C., Abood M.E., Alexander S.P.H., Di Marzo V., Elphick M.R., Greasley P.J., Hansen H.S., Kunos G., Mackie K. (2010). International union of basic and clinical pharmacology. LXXIX. Cannabinoid receptors and their ligands: Beyond CB_1_ and CB_2_. Pharmacol. Rev..

[7] Valeri A., Mazzon E. (2021). Cannabinoids and neurogenesis: The promised solution for neurodegeneration?. Molecules.

[8] Leo L.M., Abood M.E. (2021). CB1 cannabinoid receptor signaling and biased signaling. Molecules.

[9] Lu D., Immadi S.S., Wu Z., Kendall D.A. (2019). Translational potential of allosteric modulators targeting the cannabinoid CB1 receptor. Acta Pharmacol. Sin..

[10] Atalay S., Jarocka-Karpowicz I., Skrzydlewska E. (2020). Antioxidative and anti-inflammatory properties of cannabidiol. Antioxidants.

[11] Burstein S. (2015). Cannabidiol (CBD) and its analogs: A review of their effects on inflammation. Bioorg. Med. Chem..

[12] Haspula D., Clark M.A. (2020). Cannabinoid receptors: An update on cell signaling, pathophysiological roles and therapeutic opportunities in neurological, cardiovascular, and inflammatory diseases. Int. J. Mol. Sci..

[13] Tham M., Yilmaz O., Alaverdashvili M., Kelly M.E.M., Denovan-Wright E.M., Laprairie R.B. (2019). Allosteric and orthosteric pharmacology of cannabidiol and cannabidiol-dimethylheptyl at the type 1 and type 2 cannabinoid receptors. Br. J. Pharmacol..

[14] Soundara Rajan T., Giacoppo S., Scionti D., Diomede F., Grassi G., Pollastro F., Piattelli A., Bramanti P., Mazzon E., Trubiani O. (2017). Cannabidiol activates neuronal precursor genes in human gingival mesenchymal stromal cells. J. Cell. Biochem..

[15] Cashman N.R., Durham H.D., Blusztajn J.K., Oda K., Tabira T., Shaw I.T., Dahrouge S., Antel J.P. (1992). Neuroblastoma × spinal cord (NSC) hybrid cell lines resemble developing motor neurons. Dev. Dyn..

[16] Moreno-Martet M., Mestre L., Loría F., Guaza C., Fernández-Ruiz J., de Lago E. (2012). Identification of receptors and enzymes for endocannabinoids in NSC-34 cells: Relevance for in vitro studies with cannabinoids in motor neuron diseases. Neurosci. Lett..

[17] Aguado T., Palazuelos J., Monory K., Stella N., Cravatt B., Lutz B., Marsicano G., Kokaia Z., Guzmán M., Galve-Roperh I. (2006). The endocannabinoid system promotes astroglial differentiation by acting on neural progenitor cells. J. Neurosci. Off. J. Soc. Neurosci..

[18] Díaz-Alonso J., Guzmán M., Galve-Roperh I. (2012). Endocannabinoids via CB_1_ receptors act as neurogenic niche cues during cortical development. Philos. Trans. R. Soc. B Biol. Sci..

[19] Puighermanal E., Marsicano G., Busquets-Garcia A., Lutz B., Maldonado R., Ozaita A. (2009). Cannabinoid modulation of hippocampal long-term memory is mediated by mTOR signaling. Nat. Neurosci..

[20] Laun A.S., Shrader S.H., Brown K.J., Song Z.-H. (2019). GPR3, GPR6, and GPR12 as novel molecular targets: Their biological functions and interaction with cannabidiol. Acta Pharmacol. Sin..

[21] Nogueras-Ortiz C., Yudowski G.A. (2016). The multiple waves of cannabinoid 1 receptor signaling. Mol. Pharmacol..

[22] Delgado-Peraza F., Ahn K.H., Nogueras-Ortiz C., Mungrue I.N., Mackie K., Kendall D.A., Yudowski G.A. (2016). Mechanisms of biased β-arrestin-mediated signaling downstream from the cannabinoid 1 receptor. Mol. Pharmacol..

[23] Compagnucci C., Di Siena S., Bustamante M.B., Di Giacomo D., Di Tommaso M., Maccarrone M., Grimaldi P., Sette C. (2013). Type-1 (CB1) cannabinoid receptor promotes neuronal differentiation and maturation of neural stem cells. PLoS ONE.

[24] Iroegbu J.D., Ijomone O.K., Femi-Akinlosotu O.M., Ijomone O.M. (2021). ERK/MAPK signalling in the developing brain: Perturbations and consequences. Neurosci. Biobehav. Rev..

[25] Laun A.S., Song Z.H. (2017). GPR3 and GPR6, novel molecular targets for cannabidiol. Biochem. Biophys. Res. Commun..

[26] Tanaka S., Miyagi T., Dohi E., Seki T., Hide I., Sotomaru Y., Saeki Y., Antonio Chiocca E., Matsumoto M., Sakai N. (2014). Developmental expression of GPR3 in rodent cerebellar granule neurons is associated with cell survival and protects neurons from various apoptotic stimuli. Neurobiol. Dis..

[27] Landeira B.S., Santana T., Araújo J.A.M., Tabet E.I., Tannous B.A., Schroeder T., Costa M.R. (2018). Activity-independent effects of CREB on neuronal survival and differentiation during mouse cerebral cortex development. Cereb. Cortex.

[28] Cañón E., Cosgaya J.M., Scsucova S., Aranda A. (2004). Rapid effects of retinoic acid on CREB and ERK phosphorylation in neuronal cells. Mol. Biol. Cell.

[29] Mehta G., Sanyal K., Abhishek S., Rajakumara E., Ghosh S.K. (2022). Minichromosome maintenance proteins in eukaryotic chromosome segregation. BioEssays.

[30] Shivji M.K.K., Kenny M.K., Wood R.D. (1992). Proliferating cell nuclear antigen is required for DNA excision repair. Cell.

[31] Graefe C., Eichhorn L., Wurst P., Kleiner J., Heine A., Panetas I., Abdulla Z., Hoeft A., Frede S., Kurts C. (2019). Optimized Ki-67 staining in murine cells: A tool to determine cell proliferation. Mol. Biol. Rep..

[32] Fu F., Li L.S., Li R., Deng Q., Yu Q.X., Yang X., Pan M., Han J., Zhen L., Zhang L.N. (2020). All-trans-retinoid acid induces the differentiation of P19 cells into neurons involved in the PI3K/Akt/GSK3β signaling pathway. J. Cell. Biochem..

[33] Li M.-Y., Chang C.-T., Han Y.-T., Liao C.-P., Yu J.-Y., Wang T.-W. (2018). Ginkgolide B promotes neuronal differentiation through the Wnt/β-catenin pathway in neural stem cells of the postnatal mammalian subventricular zone. Sci. Rep..

[34] Grünblatt E., Bartl J., Walitza S. (2018). Methylphenidate enhances neuronal differentiation and reduces proliferation concomitant to activation of Wnt signal transduction pathways. Transl. Psychiatry.

[35] Kim K.K., Adelstein R.S., Kawamoto S. (2009). Identification of neuronal nuclei (NeuN) as Fox-3, a new member of the Fox-1 gene family of splicing factors*. J. Biol. Chem..

[36] Rosskothen-Kuhl N., Illing R.B. (2014). Gap43 transcription modulation in the adult brain depends on sensory activity and synaptic cooperation. PLoS ONE.

[37] Bustos F.J., Ampuero E., Jury N., Aguilar R., Falahi F., Toledo J., Ahumada J., Lata J., Cubillos P., Henríquez B. (2017). Epigenetic editing of the Dlg4/PSD95 gene improves cognition in aged and Alzheimer’s disease mice. Brain.

[38] Ogorodnikov A., Levin M., Tattikota S., Tokalov S., Hoque M., Scherzinger D., Marini F., Poetsch A., Binder H., Macher-Göppinger S. (2018). Transcriptome 3′end organization by PCF11 links alternative polyadenylation to formation and neuronal differentiation of neuroblastoma. Nat. Commun..

[39] Isgrò M.A., Bottoni P., Scatena R., Scatena R. (2015). Neuron-specific enolase as a biomarker: Biochemical and clinical aspects. Advances in Cancer Biomarkers: From Biochemistry to Clinic for a Critical Revision.

[40] Kojetin D.J., Burris T.P. (2014). REV-ERB and ROR nuclear receptors as drug targets. Nat. Rev. Drug Discov..

[41] Lee J.M., Kim H., Baek S.H. (2021). Unraveling the physiological roles of retinoic acid receptor-related orphan receptor α. Exp. Mol. Med..

[42] Liu H., Aramaki M., Fu Y., Forrest D., Forrest D., Tsai S. (2017). Chapter eight-Retinoid-related orphan receptor β and transcriptional control of neuronal differentiation. Current Topics in Developmental Biology.

[43] Bojesen I.N., Hansen H.S. (2005). Membrane transport of anandamide through resealed human red blood cell membranes. J. Lipid Res..

[44] Bragança V.A.N., França T.G., de Jesus A.C.S.P.S., Palheta I.C., Melo F.P.A., Neves P.A.P.F.G., Lima A.B., Borges R.S. (2020). Impact of conformational and solubility properties on psycho-activity of cannabidiol (CBD) and tetrahydrocannabinol (THC). Chem. Data Collect..

[45] Fezza F., Oddi S., Di Tommaso M., De Simone C., Rapino C., Pasquariello N., Dainese E., Finazzi-Agrò A., Maccarrone M. (2008). Characterization of biotin-anandamide, a novel tool for the visualization of anandamide accumulation. J. Lipid Res..

[46] Kaczocha M., Glaser S.T., Deutsch D.G. (2009). Identification of intracellular carriers for the endocannabinoid anandamide. Proc. Natl. Acad. Sci. USA.

[47] Oddi S., Fezza F., Pasquariello N., D’Agostino A., Catanzaro G., De Simone C., Rapino C., Finazzi-Agrò A., Maccarrone M. (2009). Molecular identification of albumin and Hsp70 as cytosolic anandamide-binding proteins. Chem. Biol..

[48] André E., Conquet F., Steinmayr M., Stratton S.C., Porciatti V., Becker-André M. (1998). Disruption of retinoid-related orphan receptor beta changes circadian behavior, causes retinal degeneration and leads to vacillans phenotype in mice. EMBO J..

[49] Fu Y., Liu H., Ng L., Kim J.-W., Hao H., Swaroop A., Forrest D. (2014). Feedback Induction of a photoreceptor-specific Isoform of retinoid-related orphan nuclear receptor β by the rod transcription factor NRL*. J. Biol. Chem..

[50] Chen X.R., Heck N., Lohof A.M., Rochefort C., Morel M.P., Wehrlé R., Doulazmi M., Marty S., Cannaya V., Avci H.X. (2013). Mature purkinje cells require the retinoic acid-related orphan receptor-α (RORα) to maintain climbing fiber mono-innervation and other adult characteristics. J. Neurosci. Off. J. Soc. Neurosci..

[51] Janmaat S., Akwa Y., Doulazmi M., Bakouche J., Gautheron V., Liere P., Eychenne B., Pianos A., Luiten P., Groothuis T. (2011). Age-related purkinje cell death is steroid dependent: RORα haplo-insufficiency impairs plasma and cerebellar steroids and Purkinje cell survival. Age.

[52] Gold D.A., Baek S.H., Schork N.J., Rose D.W., Larsen D.D., Sachs B.D., Rosenfeld M.G., Hamilton B.A. (2003). RORalpha coordinates reciprocal signaling in cerebellar development through sonic hedgehog and calcium-dependent pathways. Neuron.

[53] Taglialatela-Scafati O., Pagani A., Scala F., De Petrocellis L., Di Marzo V., Grassi G., Appendino G. (2010). Cannabimovone, a cannabinoid with a rearranged terpenoid skeleton from hemp. Eur. J. Org. Chem..

[54] Gugliandolo A., Pollastro F., Grassi G., Bramanti P., Mazzon E. (2018). In vitro model of neuroinflammation: Efficacy of cannabigerol, a non-psychoactive cannabinoid. Int. J. Mol. Sci..

[55] Silvestro S., Chiricosta L., Gugliandolo A., Pizzicannella J., Diomede F., Bramanti P., Trubiani O., Mazzon E. (2020). Extracellular vesicles derived from human gingival mesenchymal stem cells: A transcriptomic analysis. Genes.

[56] Bolger A.M., Lohse M., Usadel B. (2014). Trimmomatic: A flexible trimmer for Illumina sequence data. Bioinformatics.

[57] Dobin A., Davis C.A., Schlesinger F., Drenkow J., Zaleski C., Jha S., Batut P., Chaisson M., Gingeras T.R. (2013). STAR: Ultrafast universal RNA-seq aligner. Bioinformatics.

[58] Anders S., Pyl P.T., Huber W. (2015). HTSeq—A python framework to work with high-throughput sequencing data. Bioinformatics.

[59] Love M.I., Huber W., Anders S. (2014). Moderated estimation of fold change and dispersion for RNA-seq data with DESeq2. Genome Biol..

